# MKAN-MMI: empowering traditional medicine-microbe interaction prediction with masked graph autoencoders and KANs

**DOI:** 10.3389/fphar.2024.1484639

**Published:** 2024-10-22

**Authors:** Sheng Ye, Jue Wang, Mingmin Zhu, Sisi Yuan, Linlin Zhuo, Tiancong Chen, Jinjian Gao

**Affiliations:** ^1^ The Second Affiliated Hospital of Wenzhou Medical University, Wenzhou, Zhejiang, China; ^2^ School of Laboratory Medicine and Life Sciences, Wenzhou Medical University, Wenzhou, Zhejiang, China; ^3^ Department of Clinical Laboratory, Shandong Provincial Third Hospital, Shandong University, Jinan, Shandong, China; ^4^ Department of Bioinformatics and Genomics, University of North Carolina at Charlotte, Charlotte, NC, United States; ^5^ School of Data Science and Artificial Intelligence, Wenzhou University of Technology, Wenzhou, Zhejiang, China; ^6^ Department of Rehabilitation, The Wenzhou Third Clinical Institute Affiliated to Wenzhou Medical University, Wenzhou People's Hospital, Wenzhou Maternal and Child Health Care Hospital, Wenzhou, Zhejiang, China

**Keywords:** traditional medicine (TM), medicine-microbe interactions (MMIs), artificial intelligence models, masked graph autoencoder (mask GAE), kolmogorov-arnold networks (KANs)

## Abstract

The growing microbial resistance to traditional medicines necessitates in-depth analysis of medicine-microbe interactions (MMIs) to develop new therapeutic strategies. Widely used artificial intelligence models are limited by sparse observational data and prevalent noise, leading to over-reliance on specific data for feature extraction and reduced generalization ability. To address these limitations, we integrate Kolmogorov-Arnold Networks (KANs), independent subspaces, and collaborative decoding techniques into the masked graph autoencoder (Mask GAE) framework, creating an innovative MMI prediction model with enhanced accuracy, generalization, and interpretability. First, we apply Bernoulli distribution to randomly mask parts of the medicine-microbe graph, advancing self-supervised training and reducing noise impact. Additionally, the independent subspace technique enables graph neural networks (GNNs) to learn weights independently across different feature subspaces, enhancing feature expression. Fusing the multi-layer outputs of GNNs effectively reduces information loss caused by masking. Moreover, using KANs for advanced nonlinear mapping enhances the learnability and interpretability of weights, deepening the understanding of complex MMIs. These measures significantly enhanced the accuracy, generalization, and interpretability of our model in MMI prediction tasks. We validated our model on three public datasets with results showing that our model outperformed existing leading models. The relevant data and code are publicly accessible at: https://github.com/zhuoninnin1992/MKAN-MMI.

## 1 Introduction

Traditional medicines have historically played a crucial role in safeguarding life and health. Its primary mechanisms involve inhibiting harmful bacteria, viruses, and other microorganisms, or promoting the growth of beneficial microorganisms. Microbes, including bacteria, viruses, fungi, and protozoa, are ubiquitous on Earth and have a profound impact on human life and health (Consortium, 2012). They play crucial roles in digestion and immune processes (Flint et al., 2012; Hooper et al., 2012), produce essential vitamins (LeBlanc et al., 2013), and defend against pathogens (Buffie and Pamer, 2013). While many microbes benefit the environment and human health, some can cause disease. For instance, bacteria like *Staphylococcus aureus* and *Escherichia coli*, typically harmless in the human body, can under certain conditions cause skin infections (Liu A. et al., 2024), food poisoning (Gencay et al., 2016; Glavin, 2003), and more serious diseases. Therefore, understanding the relationship between microbes and medicines is crucial in precision medicine. Traditionally, microbial resistance has been studied through clinical observations and laboratory experiments that identify resistant strains by exposing bacteria to antibiotics and observing their survival. However, these methods are time-consuming, costly, and limited in detection range. This limitation has driven the adoption of computational methods in studying microbial resistance. Currently, the core technologies for inferring microbial resistance include systems biology and network analysis, machine learning and deep learning, and graph neural networks.

The first strategy integrates bioinformatics technologies and theories to construct and analyze biological network models, studying potential interactions between microbes and antibiotics. This approach helps scientists understand the complex regulatory mechanisms of microbial resistance. Sara Green et al. used graph theory and dynamic systems theory to simulate biological networks, gaining deeper insights into the mechanisms of microbial resistance to antibiotics (Green et al., 2018). Roberta Bardini et al. utilized a multi-level Petri net (Nets-Within-Nets, NWN) computational model to simulate the effects of various antibiotic management rules on microbial resistance (Bardini et al., 2018). Liu et al. constructed and analyzed complex network models using high-throughput multi-omics data, revealing key interactions and functions in microbial communities and mechanisms affecting community structure and resistance (Liu et al., 2021). Additionally, network topology analysis identified microbes with decisive roles in microbial networks, offering new perspectives on the functions and interactions of microbes in biological systems. Wang et al. investigated the structure and function of Cladophora’s microbial community at different life cycle stages using high-throughput 16S rRNA gene sequencing and network analysis, and analyzed the key ecological processes these communities may participate in through a functional prediction database (Wang et al., 2023b). Network-based methods employs biological network models and graph theory to assist research in deciphering the complex regulatory mechanisms underlying microbe resistance. Network topology analysis enables the identification of microbes that play pivotal roles in medicine-microbe networks, providing insights into their functions and interactions within biological systems. James et al. summarized current challenges, including incomplete data, prediction errors, noise in network analysis, and limitations in experimental verification (James and Muñoz-Muñoz, 2022). These challenges limit the broader application of network analysis-based technologies.

The second strategy leverages the similarity networks of microbes and medicines, employing machine learning (Li et al., 2021) and deep learning (Guthrie et al., 2017; Bardini et al., 2018) methods to identify potential MMIs. With significant improvements in computing performance and data storage capacity, numerous databases relevant to MMI have been established. This offers a fundamental resource for exploring new interactions between microbes and medicines through machine learning technology. For instance, Zhu et al. calculated the GIP core similarity of microbes and medicines, analyzed the chemical structure similarity of medicines, constructed similarity networks and medicine-microbe interaction networks, and employed KATZ technology to identify unknown MMIs (Zhu et al., 2019). However, the KATZ method exhibits significant limitations, including poor data adaptability, high computational complexity, and parameter sensitivity. These issues may challenge the KATZ method, particularly with sparse, large-scale, and new datasets. Consequently, HeteSim was developed. HeteSim, designed for heterogeneous networks, minimizes computation and data dependence by focusing on specific and related paths, performing well in sparse situations (Shi et al., 2014). Long et al. integrated metapath2vec with bipartite network recommendation technology and devised a biased bipartite network projection algorithm to enhance MMI prediction accuracy (Long and Luo, 2020). Zhu et al. constructed a medicine similarity matrix and applied the Laplace regularized least squares technique to identify unknown MMIs (Zhu et al., 2021). Similarity network-based methods focus on extracting similarity data from multiple sources, significantly addressing the limitations of medicine-microbe network data. Additionally, they often propose more efficient feature fusion techniques to improve the representation of medicines and microbes. However, their strong reliance on specific feature extraction may limit these methods’ adaptability.

The third strategy employs GNN technology to capture complex interactions between microbes and medicines by extracting node representations from the medicine-microbe graph. Huang et al. proposed the Graph2MDA model, based on the variational graph autoencoder (VGAE), which integrates multi-source data and network topology to accurately identify unknown MMIs (Deng et al., 2022). This marks the first application of VGAE technology to MMI prediction, achieving notable results. Tian et al. proposed the SCSMDA model, which is based on the graph convolutional network (GCN) and self-supervised learning strategy and enhances node representation using meta-path technology, yielding positive results (Tian et al., 2023). Additionally, the model incorporates contrastive learning and adaptive negative sampling strategies to further enhance performance. Long et al. introduced the EGATMDA model, leveraging GCN and the graph attention network (GAT) to extract and dynamically optimize node representations by adjusting the importance of various nodes and network types (Long et al., 2020b). Wang et al. proposed the TNRGCN model, which begins by constructing a medicine-microbe-disease heterogeneous network and then employs the relational graph convolutional network (RGCN) to identify unknown MMIs (Wang et al., 2023a). The model also utilizes principal component analysis (PCA) to extract key information from multi-source similarity data. GNN-based methods effectively capture network topology information through message propagation and update operations on medicine-microbe networks, enabling accurate prediction of potential MMIs. However, these methods typically depend on uniform and dense topological networks, which are rarely encountered in real-world scenarios. Furthermore, the initial representation of medicines and microbes often fails to significantly enhance the performance of GNN-based methods.

Despite the considerable success of current MMI inference methods based on deep learning or GNN, significant challenges remain. First, the model’s generalization ability is constrained by complex feature extractors and classifiers, with limited interpretability. Second, the observed data is vastly outnumbered by unknown drug-microbe pairs, leading to severe imbalance. Third, noisy data is inevitably introduced during the data collection process. To address these issues, we have integrated KAN, independent subspace, and collaborative encoding technologies into the Mask GAE framework to develop the new MMI prediction model MKAN-MMI. First, we mask portions of the input medicine-microbe graph to decrease the model’s noise sensitivity. Second, we employ independent subspace technology, allowing GNNs to independently learn weights within their respective feature subspaces during feature extraction. Specifically, we utilize polynomial technology to divide node features into distinct subspaces and allocate specific biases and weights to each, optimizing them independently. This avoids linear dependencies and improves the model’s adaptability to unknown data, thereby enhancing feature expression. Additionally, we collaboratively decode the outputs from multi-layer GNNs to minimize losses from masking operations. Subsequently, we incorporate KAN technology in the linear output layer to enhance weight learnability and interpretability, improving the model’s understanding of the complex interactions between microbes and medicines. These measures have significantly improved the model’s prediction accuracy, generalization ability, and interpretability. Our contributions can be summarized as follows:

1. Under the Mask GAE framework, we integrated KAN, independent subspace, and collaborative decoding technologies to develop a new MMI prediction model that achieved stable and reliable results.
2. We implemented independent subspace technology, enabling each feature subspace to independently learn weights and enhance expression capability.
3. We employed KAN technology to improve the learnability and interpretability of weights, thus enabling the model to capture detailed interactions between microbes and medicines.
4. We adopted collaborative decoding technology to integrate GNN’s multi-layer outputs, minimizing loss from masking.

## 2 Methods

The aim of this study is to identify potential MMIs among numerous unobserved medicine-microbe pairs, using observed MMIs as a basis. Since traditional biochemical or clinical experiments are often costly and time-consuming, developing efficient computational methods is crucial for rapidly identifying these unknown associations. The current research employs three main strategies: 1) integrating systems biology and network analysis, along with bioinformatics methods and mathematical modeling, to analyze the response mechanisms of microbes to drugs; 2) utilizing machine learning and deep learning techniques to extract similar features between microbes and drugs for predicting potential unknown interactions; and 3) applying GNN to extract topological features from known interactions, enhancing the representation of microbe and drug nodes. These strategies significantly enhance research efficiency for unknown MMIs and provide substantial support for understanding the complex interaction networks between microbes and drugs. However, these methods face practical challenges, particularly in terms of model generalization, which is often limited by the complexity of feature extractors and classifiers.

We integrated KAN, independent subspace, and collaborative decoding techniques into the Mask GAE framework to propose the MMI prediction model MKAN-MMI. Compared to traditional GNN-based MMI prediction models, our approach exhibits three main differences. First, we employ independent subspace technology, enabling subspaces to autonomously learn weights. This prevents weight sharing among subspaces and reduces linear correlations, thereby enhancing their expressiveness. Second, we apply collaborative decoding technology to conduct cross-Hadamard product operations on GNN’s multi-layer outputs, improving data utilization and the model’s adaptability to sparse data. Third, we utilize KAN technology to enhance the learnability and interpretability of weights, deepening our understanding of the complex interactions between medicines and microbes.

### 2.1 Model overview

**FIGURE 1:**
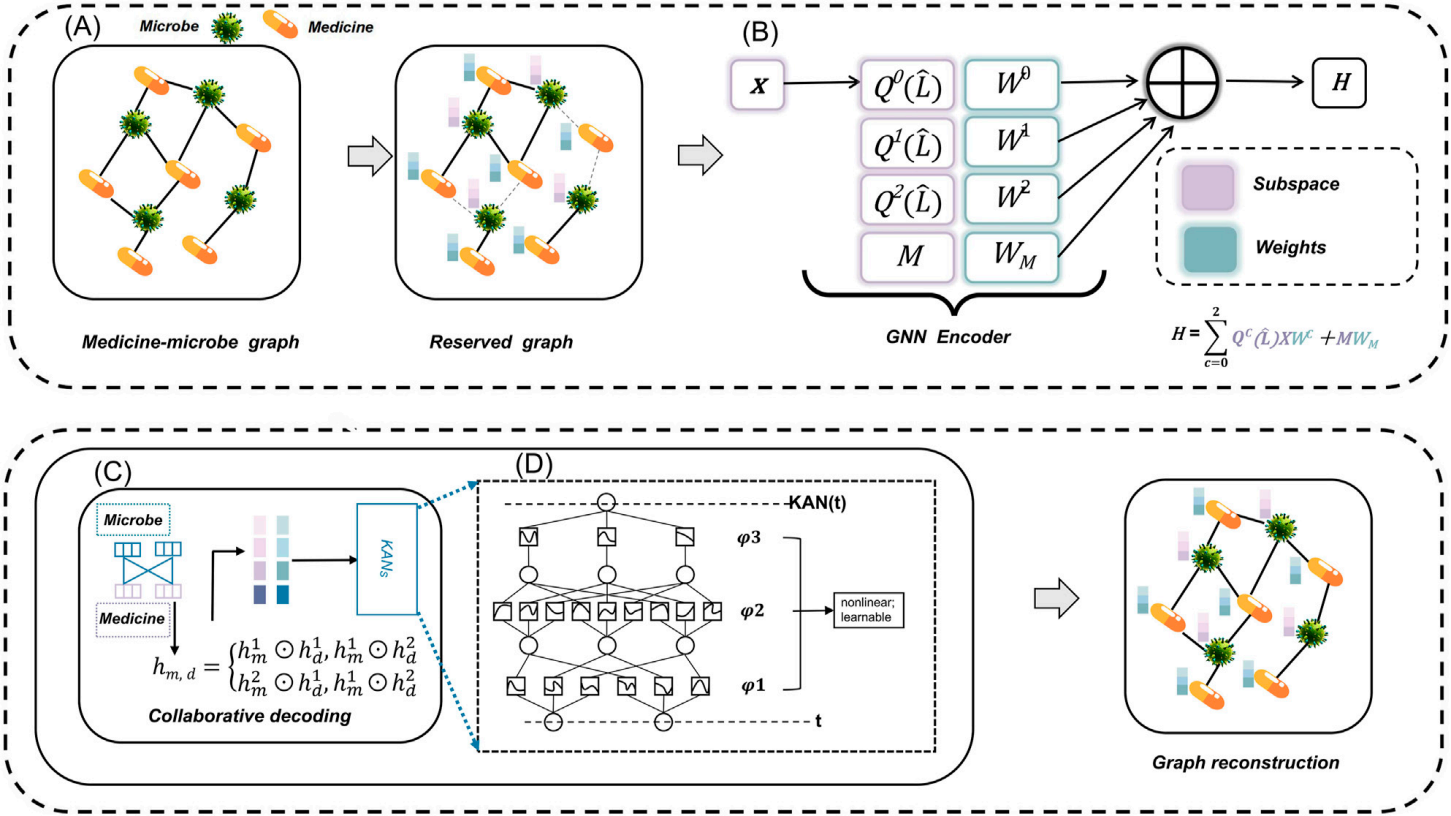
The MKAN-MMI model’s architecture comprises: **(A)** constructing and masking the medicine-microbe graph, **(B)** extracting microbe and medicine representations using independent subspace technology, **(C)** reconstructing the masked graph with collaborative decoding, and **(D)** employing KAN technology.

Figure 1 illustrates the architecture of the MKAN-MMI model. In module (A), we gather observed MMIs along with original microbe and medicine information from the database to construct the initial medicine-microbe graph. Subsequently, we mask traversed MMIs by sampling nodes, employing a random walk strategy. In module (B), independent subspace technology is applied to learn weights for each subspace independently, facilitating the extraction of multi-layer representations of microbes and medicines. In module (C), the cross-Hadamard product is applied to the multi-layer output from module (B) to produce the final medicine-microbe pair representation. Subsequently, KAN technology predicts the score and reconstructs the medicine-microbe graph. Module (D) encapsulates the operating rules of KAN.

### 2.2 Masked graph autoencoders

Recently, GAE technology has achieved significant success due to its self-supervised nature. The architecture of GAE is straightforward, comprising only two main components: a GNN encoder and a decoder. The GAE process is well-defined: the GNN encoder extracts node embeddings from the input graph, and the decoder is trained to reconstruct based on known links. The objective function is defined as Equations 1–3:

$$L_{1}=\frac{1}{E^{+}}\underset{<m,d>\in E^{+}}{\sum }logI\left(h_{m},h_{d}\right),$$ (1)

$$L_{2}=\frac{1}{E^{-}}\underset{<\hat{m},\hat{d}>\in E^{-}}{\sum }logI\left(h_{\hat{m}},h_{\hat{d}}\right),$$ (2)

$$L_{\mathrm{GAE}}=-\left(L_{1}+L_{2}\right)$$ (3)

where $L_{1}\left(L_{2}\right)$ denotes the loss on positive (negative) links, $E^{+}\left(E^{-}\right)$ represents the sets of positive (negative) links, I(⋅) is the decoder function, and $h_{m}$ and $h_{d}$ are the embeddings of nodes m and d , respectively.

Numerous studies have demonstrated that GAE exhibits enhanced performance following appropriate masking of the input graph (Devlin et al., 2019; He et al., 2022; Hou et al., 2022). Similarly, this study aims to identify potential MMIs from unknown medicine-microbe pairs using a self-supervised strategy within the Mask GAE framework. The observed medicine-microbe graph is represented as G=<V,E,X> , where V includes all microbes and medicines, E denotes the observed MMIs, and X is defined as the initial node representation. Inspired by previous research (Tan et al., 2023), we sampled the starting nodes from the observed medicine-microbe graph according to Equation 4:

$$S=Bernoulli\left(G,\gamma \right),$$ (4)

where S denotes the set of starting nodes, and γ indicates the sampling rate. Then, following the random walk rule, the MMIs traversed from the starting node are masked as Equations 5 and 6:

$$E_{\mathrm{masked}}=RandomWalk\left(S,l\right),$$ (5)

$$G_{\mathrm{reserved}}=<V_{M},V_{D},E_{\mathrm{reserved}},X>,\;E_{\mathrm{reserved}}=E-E_{\mathrm{masked}},$$ (6)

where $E_{m}ask$ denotes the masked MMIs, while the reserved MMIs are referred to as $E_{r}eserved$ . Additionally, $G_{r}eserved$ represents the reserved medicine-microbe graph. Subsequently, $G_{r}eserved$ is fed into the GNN encoder to extract embeddings of microbes and medicines.

### 2.3 Independent subspace

The GNN model accurately extracts node representations from the graph’s topological information, excelling in various graph tasks and attracting significant attention. The GNN model operates by performing multiple aggregation and update operations using the adjacency and feature matrices to extract features. Aggregation and update operations can be modified to create GNN variants suitable for various scenarios. While this model offers convenience, it also presents some challenges. Studies indicate that during the iteration of GNN models, feature subspaces are likely to exhibit approximate linear correlations (Sun et al., 2023). This significantly diminishes the subspace’s expressive power. The primary cause is the shared weights across multiple layers of feature subspaces (Sun et al., 2023). Inspired by these findings, we have integrated independent subspace technology into the Mask GAE framework to enhance the model’s feature extraction capabilities.

Specifically, we introduce a new GNN architecture centered on using polynomial technology to deshare weights in feature subspaces. According to Chebyshev’s theorem, the aggregation function is expressed as Equation 7:

$$H=\overset{C}{\underset{c=0}{\sum }}Q^{c}\left(\hat{L}\right)XW^{c},$$ (7)

where H represents the representation of microbes (medicines), $Q^{c}\left(\cdot \right)$ denotes the c -order term of a polynomial, X is the initial representation of microbes (medicines), and $W^{c}$ is the weight learned in the c -th feature subspace.

From the above equation, it is evident that the feature subspace is closely linked to the initial representation of microbes (medicines). In the medicine-microbe graph, the dimensionality of the initial representation may limit the formation of the feature space. Consequently, we apply singular value decomposition to the adjacency matrix as Equation 8:

$$M=\bar{\mathrm{UV}},\hat{A}=UV\Sigma^{T},$$ (8)

where U and V represent the principal components and its corresponding singular values, respectively. The aim of this procedure is to expand the feature subspace, thereby enhancing the data. This subspace independently learns the weight $W_{M}$ , and the aggregation function is derived as follows as Equation 9:

$$H=\overset{C}{\underset{c=0}{\sum }}Q^{c}\left(\hat{L}\right)XW^{c}+MW_{M},$$ (9)

As illustrated in Figure 1, with C equal to 2, the GNN architecture comprises four independent subspaces: $Q^{0}\left(\hat{L}\right)X$ , $Q^{1}\left(\hat{L}\right)X$ , $Q^{2}\left(\hat{L}\right)X$ , and M . This study introduces independent subspace technology to segregate these subspaces and facilitate independent weight learning within them. This approach prevents the approximate linear correlation among multiple GNN subspaces during the iteration process, thereby enhancing their expressive power.

### 2.4 Collaborative decoding

Given a microbe m and a medicine d , the GNN extracts representations htm and htd from the input graph. Typically, the current GAE framework employs cosine similarity or MLP operations during the decoding stage as Equation 10:

$$s\left(m,d\right)=<h_{m}^{C},h_{d}^{C}>\;or\;s\left(m,d\right)=MLP\left(h_{m}^{C}∥h_{d}^{C}\right),$$ (10)

where s represents the predicted score. While these methods appear simple, they are effective. However, applying these operations presents challenges for MMI prediction tasks. The primary challenges are twofold. First, within the Mask GAE framework, masking some MMI may result in the loss of crucial topological information. Second, in the medicine-microbe dataset, the number of observed MMI is significantly lower than that of unknown pairs, contributing to data sparsity.

Research indicates that connecting, adding, or multiplying the multi-layer outputs of GNNs can enhance the data processing. However, a major drawback of this strategy is the introduction of significant noise, which impacts the final microbe (medicine) representation. Drawing inspiration from prior research (Tan et al., 2023), we have implemented collaborative decoding technology and the cross-Hadamard product to integrate representations of microbes and medicines across each GNN layers as Equation 11:

$$h_{<m,d>}={∥}_{a,b=1}^{L}h_{m}^{a}⊙h_{d}^{b},$$ (11)

where $h_{<m,d>}$ denotes the final representation of the medicine-microbe pair <m,d> . The terms ⊙ and ∥ refer to the “Hadamard product” and “concatenation” operations, and L indicates the number of encoder layers. The cross-Hadamard product operation effectively integrates multi-layer information, promoting similarity while reducing differentiation between microbes and medicines. This ensures that the output representation of the medicine-microbe pair retains essential information.

### 2.5 Kolmogorov-arnold networks (KANs)

MLP is capable of describing nonlinear functions; its simplicity and feasibility have made it the most popular neural network currently. The core of MLP involves performing linear mapping on the input, often incorporating nonlinear activation functions. MLP has been integrated into various network architectures, including GNNs and convolutional neural networks. However, recent studies have highlighted significant challenges facing MLP that cannot be ignored. For instance, MLP often requires stacking, and typically has a large parameter scale. Moreover, MLP’s functionality relies entirely on the interplay of neurons, resulting in limited interpretability. Initially, MLP places the activation function at the neuron level, whereas KAN applies univariate and spline functions to the weights. This approach allows KAN to improve weight learnability and interpretability. KAN theory originates from the concept that multivariate continuous functions can be derived by combining univariate functions via binary addition, as Equation 12:

$$f\left(t\right)=f\left(t_{1},t_{2},\ldots ,t_{n}\right)=\overset{2n+1}{\underset{v=1}{\sum }}\varphi_{v}\left(\overset{n}{\underset{u=1}{\sum }}\varphi_{v,u}\left(t_{u}\right)\right),$$ (12)

where $t_{1},t_{2},\ldots ,t_{n}$ are binary univariate variables; $\varphi_{v,u}$ are binary functions, where n represents the number of neurons, and $\varphi_{v}$ is a real function. However, some studies have indicated that these univariate functions are occasionally non-smooth, limiting the applicability of KAN theory (Poggio et al., 2020; Girosi and Poggio, 1989). Fortunately, Liu et al. have observed that functions commonly used in daily life are smooth, reigniting interest in KAN theory (Liu Z. et al., 2024).

To achieve arbitrary depth with KAN, a straightforward approach is the integration of MLP with KAN theory, as Equation 13:

$$KAN\left(t\right)=\left(\varphi_{K-1}◦\varphi_{K-2}\ldots \varphi_{1}◦\varphi_{0}\right)t,$$ (13)

where K denotes the number of KAN layers.

As depicted in Figure 1B, the MKAN-MMI model employs KAN to process the final representation of the medicine-microbe pair for predicting the final score. Subsequent experiments demonstrate that integrating KAN technology significantly enhances the model’s prediction performance.

## 3 Results

### 3.1 Data preparation

To verify the accuracy of the MKAN-MMI model in MMI prediction, we conducted evaluations across several publicly accessible MMI databases. Drawing on prior research (Long et al., 2020a), we selected three databases for evaluation: MDAD (Sun et al., 2018), DrugVirus (Andersen et al., 2020), and aBiofilm (Long et al., 2020a). The MDAD database comprises 1,373 medicines, 173 microbes, and 2,470 MMI in total. The aBiofilm database includes 1,720 medicines, 140 microbes, and 2,884 MMIs in total. The DrugVirus database contains 175 medicines, 95 microbes, and 933 MMIs in total.

Additionally, we gathered similarity data for microbes and medicines from previous studies (Tian et al., 2023). For microbes, we gathered functional similarity $\left(F_{\mathrm{1MS}}\right)$ and Gaussian interaction kernel similarity $\left(GIP_{\mathrm{1MS}}\right)$ data. Detailed calculations for $F_{\mathrm{1MS}}$ are available in the works of Kamneva (2017). The calculation of $GIP_{\mathrm{1MS}}$ and the integration of $F_{\mathrm{1MS}}$ and $GIP_{\mathrm{1MS}}$ are discussed in previous work (Tian et al., 2023). For medicines, we collected structural similarity $\left(F_{\mathrm{2MS}}\right)$ and Gaussian interaction kernel similarity $\left(GIP_{\mathrm{2MS}}\right)$ data. Hattori’s work (Hattori et al., 2010) details the calculation of $F_{\mathrm{2MS}}$ , while the process of calculating $GIP_{\mathrm{2MS}}$ and integrating it with $F_{\mathrm{2MS}}$ can be found in earlier studies (Tian et al., 2023). We used the integrated similarity features of microorganisms and drugs as the model’s initial representation. Negative samples were generated via random sampling. Known MMIs were treated as positive samples, while the remaining unknown drug-disease pairs were considered negative samples.

### 3.2 Experiment setting

We compared the MKAN-MMI model against eight models, encompassing classic GNN models such as GCN (Li et al., 2018) and GAT (Veličković et al., 2018), as well as advanced models like DTI-CNN (Peng et al., 2020), NIMCGCN (Li et al., 2020), MMGCN (Tang et al., 2021), and DTIGAT (Wang et al., 2021), Graph2MDA (Deng et al., 2022), SCSMDA (Tian et al., 2023), and GCNMDA (Devlin et al., 2019). Notably, DTI-CNN (Peng et al., 2020), NIMCGCN (Li et al., 2020), MMGCN (Tang et al., 2021), and DTI-GAT (Wang et al., 2021) were not originally designed for MMI prediction tasks. Consequently, these models required modifications, including adjusting the input to the initial representation of microbes and medicines, and to the medicine-microbe graphs. To ensure fairness, the study maintained a consistent data partitioning ratio and conducted uniform 5-fold cross-validation across all experiments. The proposed MKAN-MMI model primarily considers the root node sampling rate, random walk length, and number of feature subspaces. Empirically, these parameters are set to 0.5, 3, and 3 by default. The training-to-test set ratio is set to 4:1, and the positive-to-negative sample ratio is also set to 1:1. The primary evaluation metrics employed were AUC (area under the ROC curve) and AUPR (area under the precision-recall curve). Additionally, for a comprehensive assessment, accuracy (ACC), precision (PRE), F1-score, and Matthews correlation coefficient (MCC) served as auxiliary metrics, similar to previous practice (Zhou et al., 2024; Wei et al., 2024; Wang et al., 2024; Ma et al., 2024; Xu et al., 2023).

### 3.3 Performance comparison

Under identical data partitioning conditions, we assessed the performance of the proposed model alongside that of existing comparison models. Table 1 displays the AUC and AUPR performance metrics of all models across the MDAD, DrugVirus, and aBiofilm datasets. Significantly, the MKAN-MMI model achieved the highest performance in both AUC and AUPR metrics across all datasets, ranking first. The SCSMDA model followed closely, securing the second rank. The DTI-CNN model, ranking third in the AUC metric, underscored the autoencoder’s advantage in feature extraction. However, it exhibited slightly inferior performance in the AUPR metric within the DrugVirus and aBiofilm datasets. The underperformance of other GNN models highlights the challenges of strategies relying solely on observed MMIs. GNN model designs heavily depend on network topology during aggregation and updates, often neglecting the nodes’ initial representations. Specifically, in the MDAD, DrugVirus, and aBiofilm datasets, the observed MMIs are significantly fewer than the unknown medicine-microbe pairs, suggesting the initial representation could play a crucial role in MMI prediction.

**TABLE 1:** Comparison of MKAN-MMI with other outstanding models (%).

Models/Datasets metrics	MDAD AUC	AUPR	DrugVirus AUC	AUPR	aBiofilm AUC	AUPR
GCN Li et al. (2018)	86.85	87.35	81.36	79.61	89.51	89.91
GAT Veličković et al. (2018)	87.78	88.68	81.80	80.01	90.37	89.60
DTI-GAT Wang et al. (2021)	89.56	90.12	78.73	79.32	85.15	87.16
NIMCGCN Li et al. (2020)	90.53	91.47	84.65	84.62	91.48	92.31
NMGCN Tang et al. (2021)	89.38	90.61	78.69	76.64	90.81	91.71
DTI-CNN Peng et al. (2020)	93.32	92.63	84.90	83.3	94.67	94.14
Graph2MDA Deng et al. (2022)	87.22	90.93	77.14	79.48	92.75	94.85
GCNMDA Devlin et al. (2019)	91.79	90.38	83.11	79.45	94.14	93.29
SCSMDA Tian et al. (2023)	95.76	94.76	88.81	86.30	96.39	95.39
MKAN-MMI	99.58	99.60	94.54	92.32	99.50	99.63

The results of the comparison method are sourced from prior studies (Tian et al., 2023).

The SCSMDA model utilizes GCN technology and self-supervised learning strategies, incorporating meta-path and graph contrast learning techniques to enhance node representations, resulting in positive outcomes. However, the increased complexity of its architecture may hinder the model’s generalization. The proposed model adopts the encoder-decoder framework of GAE to reconstruct the medicine-microbe graph, demonstrating greater efficiency and accuracy in identifying unknown MMIs compared to the SCSMDA model. This improvement can be attributed to several factors. First, independent subspace technology is employed to enhance subspace representation capabilities. Second, collaborative decoding technology integrates multi-layer GNN outputs to improve node representations of medicines and microbes. Finally, the proposed model applies KAN technology to enhance its flexibility and generalization capabilities.

### 3.4 Parameter experiments

The proposed model incorporates several customizable parameters, including GNN encoder type, node sampling rate, and masked path length. We assessed the impact of different parameter settings on the MKAN-MMI model’s performance across three databases, confirming its adaptability to these parameters.

#### 3.4.1 Node sampling rate analysis

**FIGURE 2:**
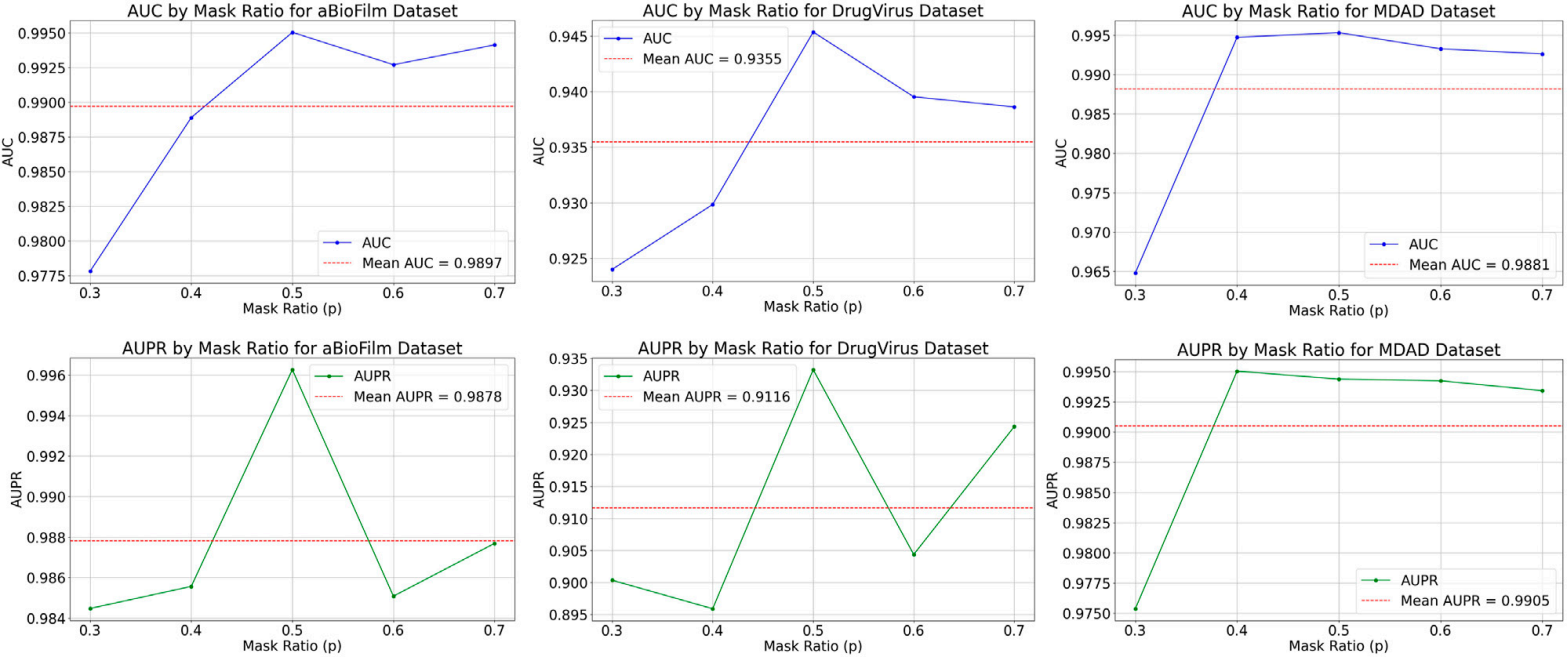
Results of MKAN-MMI model using different node sampling rates.

The MKAN-MMI model offers a broad spectrum of node sampling rate settings to accommodate data of varying densities. Typically, dense data necessitates a higher sampling rate to mitigate overfitting, whereas sparse data benefits from a lower rate to minimize information loss. Prior to inputting the medicine-microbe graph into the MKAN-MMI model, we sampled nodes using a Bernoulli distribution at rates between 0.3 and 0.7. From the selected nodes, masked paths (MMIs) are established using a random walk strategy. In the experiments, the path length was consistently set to 3. Figure 2 displays the results, showing that the model’s performance improves with higher node sampling rates. This suggests that suitably masking observed MMIs can alleviate issues related to overfitting or noise. However, excessive sampling of nodes results in a correspondingly higher number of masked MMIs. The results indicate a noticeable decline in model performance. This suggests that excessive masking of key nodes or MMIs contributes to performance degradation.

#### 3.4.2 Walk length analysis

**FIGURE 3:**
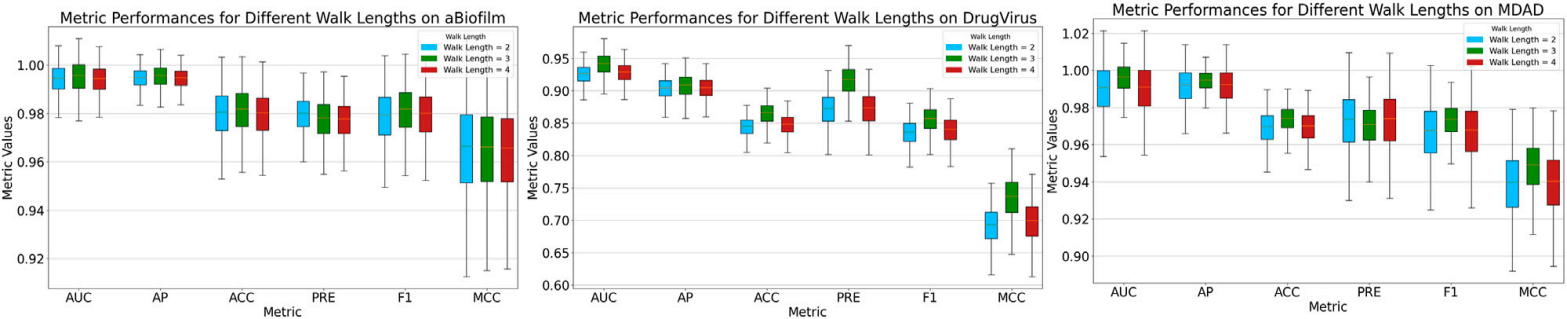
Results of MKAN-MMI model using different walk lengths.

After sampling nodes at a predefined ratio, we mask the MMIs starting from these nodes using a pre-set length dictated by the random walk strategy. The MKAN-MMI model accommodates custom walk lengths to suit various data types. In the experiments, the sampling rate was consistently maintained at 0.5. Figure 3 illustrates that the model’s overall performance exhibits minimal fluctuations. The model achieves optimal performance when the walk length is set to 3. We deduce that the model’s performance is correlated with the walk length. A shorter masking length, such as 2, may lead to fewer masked MMIs, potentially limiting the model’s training effectiveness. Conversely, a longer masking length, such as 4, could result in more masked MMIs, risking significant loss of key information.

#### 3.4.3 GNN encoder analysis

**FIGURE 4:**
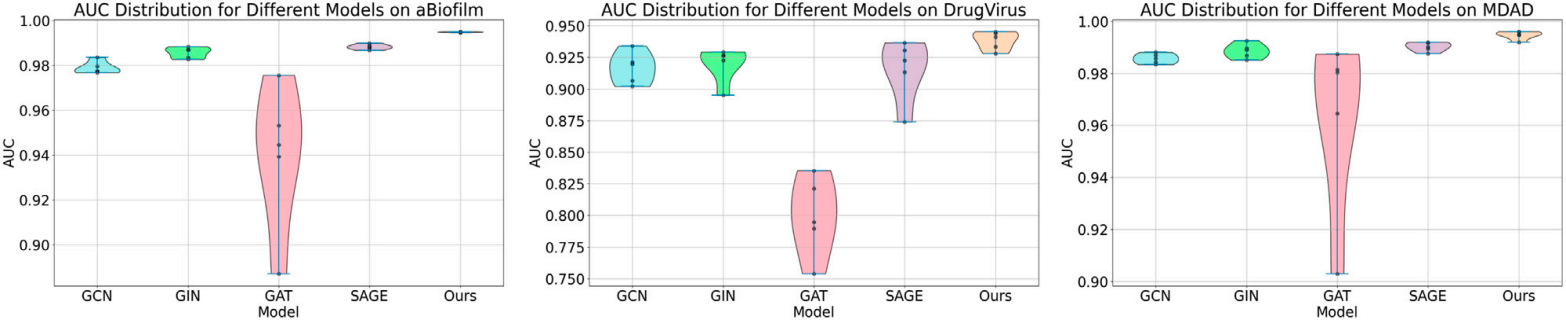
Results of MKAN-MMI model using different GNN encoders.

Within the Mask GAE framework, the GNN encoder can be customized. In our experiments, we evaluated the performance of various GNN encoders integrated into the model. Figure 4 demonstrates that encoders based on independent subspaces significantly outperform other GNN models. Additionally, the GCN, GIN, and SAGE models generally outperform the GAT model. This could be attributed to GAT’s focus on edge weight information, which may be significantly lost when masking MMIs. These traditional GNN models employ a weight-sharing mechanism that leads to linear correlations among subspaces during iterations, severely constraining their expressive capabilities. We have integrated independent subspace technology into the model to enhance the autonomous learning capabilities of subspaces, thereby boosting model performance.

#### 3.4.4 Feature subspace number analysis

**FIGURE 5:**
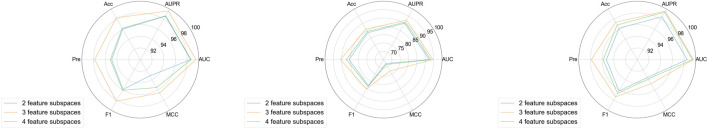
Results of MKAN-MMI model using different subsapce numbers.

Another key parameter in this study is the number of feature subspaces. We conducted experiments to explore the impact of this parameter on model performance. In these experiments, the sampling rate was fixed at 0.5 and the walk length at 3. As shown in Figure 5, the model’s overall performance exhibited little fluctuation on aBiofilm, DrugVirus, and MDAD datasets, sequentially. When the number of feature subspaces was set to 3, the model achieved optimal performance. We infer that the model’s performance is correlated with the number of feature subspaces. A smaller number, such as 2, may result in insufficient feature extraction, limiting the model’s training effectiveness, while a larger number, such as 4, may introduce redundant information, reducing performance.

### 3.5 Ablation study

We anticipate that the proposed model will excel in the MMI prediction task, primarily due to the integration of independent subspace, collaborative decoding, and KAN techniques within the Mask GAE framework. To test this hypothesis, we conducted multiple experimental series on the MDAD database. Table 2 displays the outcomes of these experiments. “GCN” and “DG” signify that the MKAN-MMI model’s encoder employs GCN and independent subspace techniques, respectively. “CD” denotes that the MKAN-MMI model’s decoder utilizes collaborative decoding technology. “ID” represents that the model extracts the output of the last layer of the microbe and medicine, performs the Hadamard product, and predicts the medicine-microbe pair score. “KAN” and “MLP” show that the MKAN-MMI model employs KAN and MLP, respectively, to predict the medicine-microbe pair score. Observations reveal that the absence of independent subspace, collaborative decoding, or KAN technology in the MKAN-MMI model leads to reduced performance. This indicates that all three technologies contribute significantly to enhancing MMI prediction. The model’s performance is poorest when it lacks collaborative decoding technology. This suggests that collaborative decoding technology effectively mitigates data sparsity issues, thereby enhancing the model’s robustness. Performance slightly declines when the model operates without independent subspace technology. Performance significantly deteriorates when the model employs MLP technology in place of KAN.

**TABLE 2:** Results of ablation study (%).

GCN	SG	CD	ID	KAN	MLP	AUC	AUPR
	√		√	√		96.67	97.63
√		√		√		98.85	98.74
	√	√		√		99.58	99.60
	√	√			√	97.77	97.18

### 3.6 AUC-based statistical significance analysis

**FIGURE 6:**
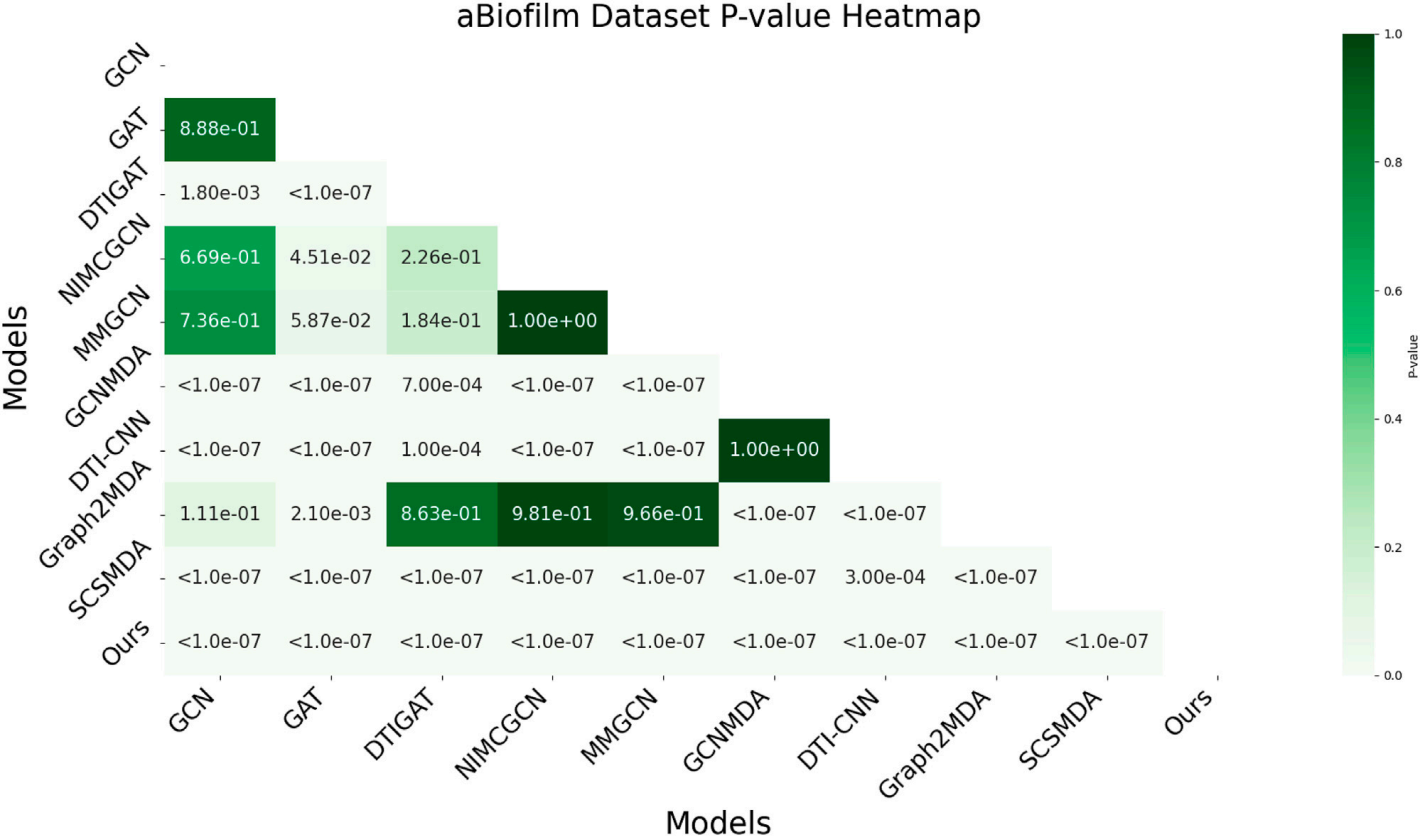
AUC-based statistical significance analysis on aBiofilm dataset.

**FIGURE 7:**
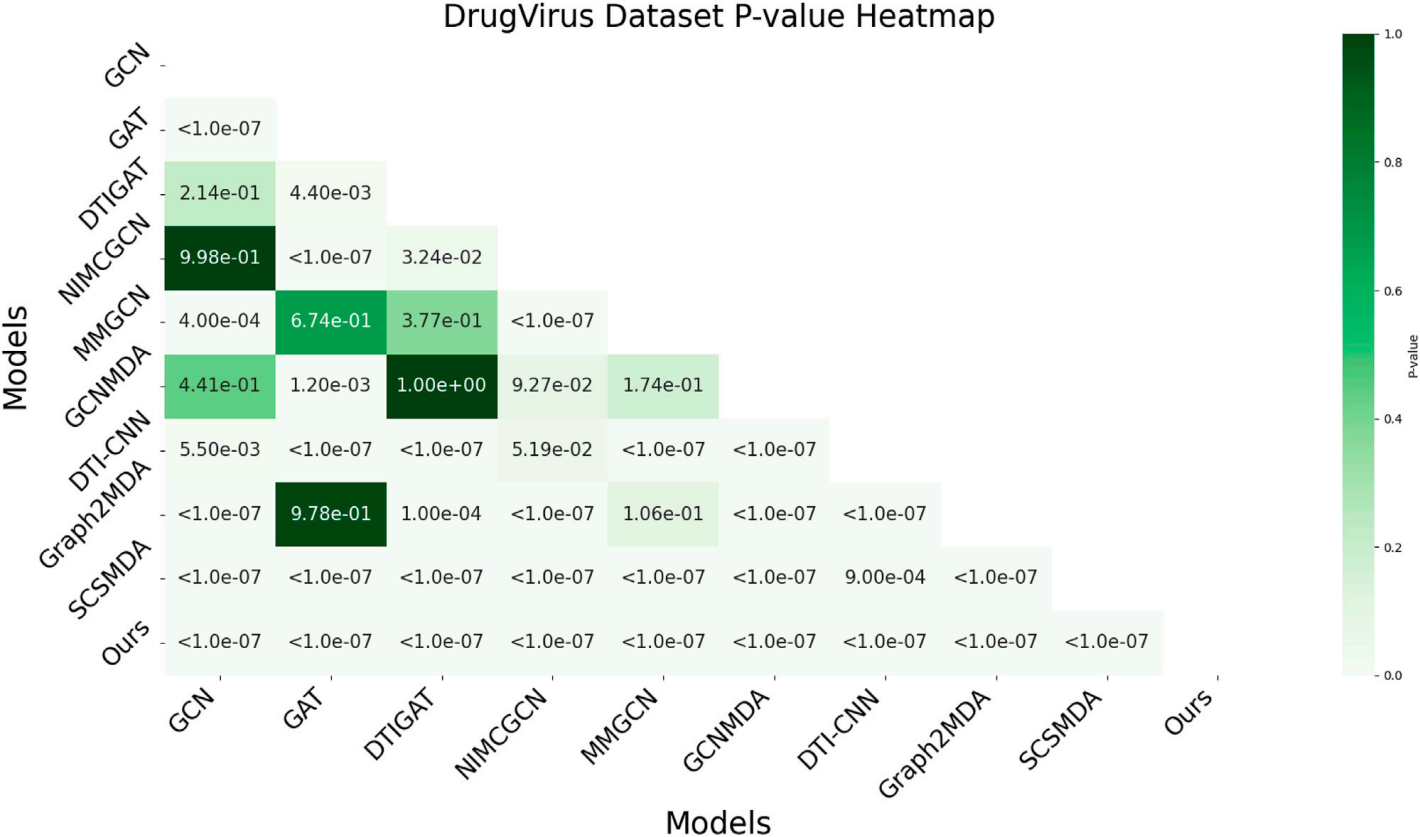
AUC-based statistical significance analysis on DrugVirus dataset.

**FIGURE 8:**
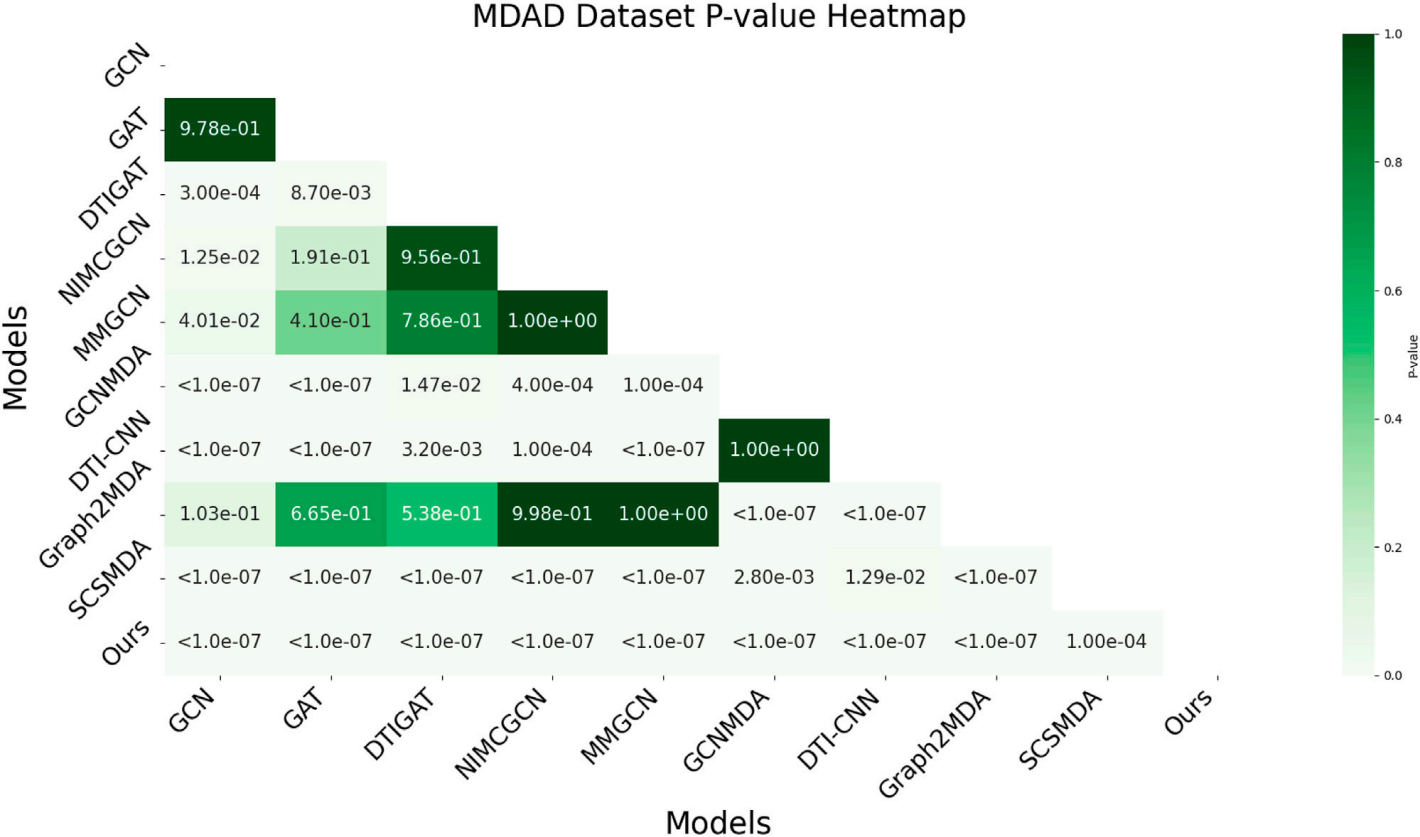
AUC-based statistical significance analysis on MDAD dataset.

In this study, we employed one-way analysis of variance (ANOVA) (St and Wold, 1989) to systematically assess whether significant differences exist in the AUC performance of various MMI prediction models across the aBiofilm, DrugVirus, and MDAD datasets, as shown in Figures 6–8, respectively. The results indicate that on the MDAD dataset, our model demonstrates a significant advantage, with *p*-values below 1.0e-07 compared to most models, underscoring its statistical significance. Notably, when compared to the Graph2MDA and DTI-CNN models, our model achieved *p*-values of 1.00e-04 and 3.20e-03, respectively. While the differences are subtle, they remain statistically significant. On the DrugVirus dataset, our model also showed significance in most comparisons. However, when compared to the NIMCGCN model, the two models performed similarly, with *p*-values as high as 0.99, indicating their comparable predictive ability on this dataset. Nonetheless, in other comparisons, our model consistently demonstrates significant superiority, with *p*-values mostly below 1.0e-07. On the aBiofilm dataset, our model also maintains a significant performance advantage. In comparisons with NIMCGCN and Graph2MDA, *p*-values were 0.66 and 0.98, respectively, indicating that our model performs similarly to these models on some evaluation metrics. Overall, the *p*-values between our model and all comparison models remain well below the significance threshold of 0.05, further validating its superior performance.

The analysis results not only confirm the performance advantage of our model but also emphasize its stability and reliability across various data environments. Additionally, the results strongly support the application value of decoupled representation learning and multi-scale fusion technology in enhancing model generalization and addressing complex interaction prediction challenges.

### 3.7 Performance evaluation

This study employed a five-fold cross-validation method to assess the performance of the MKAN-MMI model across the MDAD, DrugVirus, and aBioFilm databases. As detailed in Table 3, the proposed model demonstrated stable performance on the MMI prediction task, surpassing the current state-of-the-art SCSMDA model. Specifically, the proposed model achieved an average AUC of 99.58% on the MDAD dataset, which is 4.43% higher than SCSMDA’s 95.15%. On the DrugVirus dataset, the proposed model recorded an average AUC of 94.54%, 0.87% higher than SCSMDA’s 93.67%. On the aBioFilm dataset, our model excelled with an average AUC of 99.50%, marking an increase of 1.27% over SCSMDA’s 98.23%. Additionally, for the AUPR metric, the proposed MKAN-MMI model significantly outperforms the SCSMDA model. These results underscore the model’s effectiveness and its robust generalization capability across various settings.

**TABLE 3:** Results of ablation study (%).

Datasets/Metrics	Folds	AUC	AUPR	ACC	PRE	F1	MCC
MDAD	1	99.69	99.69	96.40	98.13	96.33	92.85
	2	99.48	99.38	97.96	97.76	97.96	95.92
	3	99.75	99.75	97.49	97.75	97.47	94.97
	4	99.41	99.44	96.82	96.10	96.85	93.37
	5	99.57	99.73	98.28	99.53	98.25	96.59
	Average	99.58	99.60	97.39	97.85	97.37	94.74
DrugVirus	1	94.62	92.24	86.22	91.18	85.23	73.10
	2	94.55	92.38	86.42	90.75	85.65	73.50
	3	94.3	92.26	85.43	89.47	84.65	71.24
	4	94.62	92.34	86.02	91.03	85.12	72.59
	5	94.59	92.39	86.61	91.15	85.83	73.68
	Average	94.54	92.32	86.14	90.72	85.30	72.82
aBIoFIlm	1	97.89	98.56	95.87	97.03	95.59	91.91
	2	99.98	99.98	99.19	98.52	99.20	98.40
	3	99.81	99.77	98.71	97.55	98.72	97.46
	4	99.86	99.84	98.64	97.87	98.65	97.30
	5	99.98	99.98	98.97	98.11	98.98	97.95
	Average	99.50	99.63	98.28	97.82	98.23	96.60

### 3.8 Case analysis

We conducted a series of case studies to assess the model’s performance under isolation. We chose the medicine Berberine from the DrugVirus database for analysis and validation. Berberine, an alkaloid derived from plants like Coptis chinensis and Phellodendron chinense, has been used historically to treat various diseases (Song et al., 2020). This medicine exhibits multiple biological activities, including antimicrobial, anti-inflammatory, antioxidant properties, and the regulation of blood sugar and lipids. Berberine inhibits protein synthesis in microbial cells, reduces inflammatory factor expression, enhances the antioxidant enzyme system, and activates AMP-activated protein kinase (AMPK). Consequently, it holds potential for treating type 2 diabetes, cardiovascular diseases, and gastrointestinal disorders. Although considered relatively safe, Berberine may interact with specific microbes. Thus, studying Berberine and its associated microbes is essential.

Epstein–Barr virus (EBV) is a γ -herpesvirus prevalent across various human populations (Young and Rickinson, 2004). EBV exhibits tumorigenic properties and is implicated in diseases like infectious mononucleosis and Hodgkin’s disease, which affect ENT regions such as the throat and lymph nodes (Macsween and Crawford, 2003). Cohen et al. explored EBV-associated lymphoproliferative diseases, including aggressive T-cell and NK-cell diseases that may impact the nasal cavity and other ENT regions, as well as ENT-related conditions like vesiculoderma-like lymphoma (Macsween and Crawford, 2003). Green et al. highlighted the role of EBV in post-transplant lymphoproliferative diseases, particularly in ENT areas like the oropharynx, with clinical presentations ranging from asymptomatic infections to aggressive lymphomas in solid organ transplant recipients (Cohen et al., 2009). Thus, investigating EBV-related drugs may aid in developing new ENT-related therapeutic strategies.

Specifically, we excluded Berberine, EBV and their associated MMIs from the dataset during model training. Subsequently, the trained model predicted the likelihood of interactions between all microbes (medicines) and Berberine (EBV). Following analysis, the top 10 microbes were identified, with results detailed in Table 4. It was confirmed that nine microbes interact with Berberine, as documented in the DrugVirus database. The results in Table 5 indicate that all 10 medicines predicted by the trained model interact with EBV and have been verified in DrugVirus. Therefore, the proposed model is demonstrably effective in independently identifying potential MMIs.

**TABLE 4:** The top 10 predicted microbes interacted with Berberine with the highest scores.

Microbes	DrugVirus	Microbes	DrugVirus
Chikungunya virus	confirmed	Human papillomavirus	confirmed
Cytomegalovirus	confirmed	Herpes simplex virus 1	confirmed
Influenza A virus	confirmed	Respiratory syncytial virus	confirmed
Hepatitis C virus	confirmed	Enterovirus A	confirmed
Sindbis virus	confirmed	Hendra virus	unconfirmed

**TABLE 5:** The top 10 predicted medicines interacted with EBV with the highest scores.

Medicines	DrugVirus	Medicines	DrugVirus
Camptothecin	confirmed	Foscarnet	confirmed
Cidofovir	confirmed	Chlorpromazine	confirmed
Artesunate	confirmed	N-MCT	confirmed
Filociclovir	confirmed	Ganciclovir	confirmed
Luteolin	confirmed	Novobiocin	confirmed

## 4 Conclusion

Microbes, existing in diverse forms across plants and animals, are integral to numerous life processes. Accurate identification of potential MMIs facilitates exploration of medicine resistance and side effects, and aids in developing new treatment strategies. This study examined various MMI prediction models and identified their key challenges. For instance, the often sparse and noisy observational data causes these models to overly rely on complex feature extraction, rendering them susceptible to overfitting and other issues. Consequently, we integrated independent subspaces, collaborative decoding, and KAN technologies into the Mask GAE framework, resulting in the proposed MMI prediction model, MKAN-MMI. Operating under the Mask GAE framework, this model mitigates the risks of overfitting and noise via masking rules. Simultaneously, the model employs independent subspace technology to prevent asymptotic correlation among subspaces, thereby enhancing their expressiveness. Furthermore, the model utilizes collaborative decoding technology to mitigate the impact of data sparsity. A series of designed experiments demonstrated the effectiveness of these measures in MMI prediction. Additionally, these results indicate that the proposed MKAN-MMI model is likely to be a valuable tool in studying microbes and medicines.

However, the proposed model faces challenges that cannot be overlooked. First, the known MMI data are too limited and highly imbalanced compared to the unknown medicine-microbe pairs. Second, there is currently no effective method to characterize microbes and medicines. Third, significant differences may exist between the newly generated data and the original dataset. To overcome these challenges, we propose the following approaches. First, leveraging large language models or pre-trained models to learn general knowledge about drugs and microorganisms to enhance node representation. Second, incorporating text descriptions, such as properties and functions of medicines and microbes, and multimodal methods like SMILES sequences, to integrate information. Third, applying transfer learning to capture the differences between new and old data, thereby improving model adaptability.

## Data Availability

The original contributions presented in the study are included in the article/supplementary material, further inquiries can be directed to the corresponding authors.
